# How to Reliably Measure Stroke Volume Index in Pulmonary Arterial Hypertension: A Comparison of Thermodilution, Direct and Indirect Fick, and Cardiac MRI

**DOI:** 10.3390/life15010054

**Published:** 2025-01-03

**Authors:** Andrea Baccelli, Deepa Gopalan, Rachel J. Davies, Gulammehdi Haji, Wendy Gin-Sing, Luke S. Howard, Francesco Lo Giudice

**Affiliations:** 1 Department of Respiratory Medicine, Royal Brompton Hospital, Guy’s and St Thomas’ NHS Foundation Trust, London SE1 7EH, UK; 2 Department of Radiology, Hammersmith Hospital, Imperial College Healthcare NHS Trust, London W12 0HS, UK; 3 National Pulmonary Hypertension Service, Hammersmith Hospital, Imperial College NHS Trust, DuCane Road, London W12 0HS, UK; 4 National Heart & Lung Institute, Imperial College London, London SW7 2AZ, UK

**Keywords:** stroke volume index, pulmonary arterial hypertension, thermodilution, direct Fick, indirect Fick, cardiac magnetic resonance

## Abstract

Background. Stroke volume index (SVI) is an important prognostic parameter in pulmonary arterial hypertension (PAH). The direct Fick (DF) method represents the gold standard for measuring it. Indirect Fick (IF) and thermodilution (TD) are simpler and widely used alternatives. However, data on the accuracy of these methods in estimating SVI in PAH are scant. We aimed to compare these different invasive methods, and in a subgroup of patients, to a non-invasive method using MRI. Methods. We enrolled 103 PAH patients undergoing a diagnostic or follow-up right heart catheterization at our centre (mean age 56 years, 56% female). The Bland–Altman analysis was used to assess agreement between methods. Potential demographic, clinical, and hemodynamic biases were explored. The accuracy of cardiac magnetic resonance (CMR)-derived SVI was assessed in a subset of patients. Results. The mean bias for IF-SVI vs. DF-SVI was −5.53 mL/min/m^2^ with a median percentage error (PE) of 15%. The mean bias was lower, 0.09 mL/min/m^2^, for TD-SVI vs. DF-SVI with a median PE of 10%. Low cardiac index and severe tricuspid regurgitation (TR) were associated with a greater bias between TD and DF. CMR-SVI showed good accuracy and precision even in patients with severe TR, compared to DF. Conclusions. The indirect Fick is the less reliable method to assess SVI also in PAH patients. Thermodilution is a valid alternative to direct Fick, but it should be used with caution in patients with severe TR or low cardiac index. SVI measured by cardiac MRI is a promising non-invasive alternative, especially in patients with severe TR. Our observation needs to be confirmed by other series and larger studies.

## 1. Introduction

Pulmonary arterial hypertension (PAH) is characterized by obstructive vascular lesions primarily affecting the small pulmonary arteries. This leads to raised pulmonary vascular resistance and increased right ventricular (RV) afterload that eventually results in right ventricular to pulmonary arterial uncoupling and right heart dysfunction and failure [1].

Hemodynamic variables reflecting RV function are important for the multi-parametric risk assessment in PAH, both at the time of diagnosis and follow-up [1]. Stroke volume index (SVI) has emerged as an independent prognostic marker, able to identify patients with a poorer outcome even when the cardiac index (CI) is in the low-risk range (i.e., ≥2.5 L/min/m^2^) [2]. SVI and mixed venous oxygen saturation (SvO_2_) have been recently mentioned as variables to further refine prognostication in patients with intermediate-risk PH in the 7th World Symposium of Pulmonary Hypertension [3,4].

Between the hemodynamic variables proven to be useful for risk stratification, right atrial pressure (RAP) and SvO_2_ are direct measures, compared to the cardiac output (CO) and the derived parameters SVI and CI that are estimated [5,6].

There are three methods to estimate the CO. The direct Fick (DF) is considered the gold standard, but it is not widely used because it requires oxygen consumption (VO_2_) measurement at the time of the procedure [5,6,7,8]. The indirect Fick uses an estimated VO_2_ using specific equations, and the Dehmer seems the most accurate [9].

Thermodilution (TD) estimates the CO by injecting a known volume of saline, colder than circulating blood, in the proximal port of a Swan–Ganz catheter positioned in the pulmonary artery. This method does not require VO_2_ measurement, but its accuracy can be influenced by factors such as severe tricuspid regurgitation (TR) and low output status, both relatively common conditions in PAH patients [7,8,10,11].

The indirect Fick (IF) method has proved to be less accurate than TD also in pulmonary arterial hypertension [7].

SVI is derived by the CO, dividing it by the heart rate, and then indexed by the body surface area.

CO and SVI can be estimated non-invasively by cardiac magnetic resonance imaging (CMR), and the methods using the left ventricular volumes and aortic flow are the most accurate [12,13].

Comparative studies between TD, IF, and DF have been published but with conflicting results, and data for SVI measures are lacking [7,8,9,14,15,16,17,18,19,20]. The aims of our study were primarily to assess the accuracy and precision of SVI assessed by IF and TD compared to the gold-standard DF in patients with PAH. Secondly, to assess the potential clinical implication of the bias between these methods. Thirdly, to investigate which demographic and hemodynamic factors are associated with a greater bias between measures. Finally, in a subgroup of patients with a CMR performed within 24 h from the right heart catheterization (RHC), we compared SVI assessed with invasive and non-invasive methods.

## 2. Methods

### 2.1. Patients

Data from all consecutive patients diagnosed with PAH between September 2022 and April 2023 at our centre were prospectively entered into the TRIPHIC database and analyzed under the local ethics committee approval number 17/LO/0563.

Patients were selected based on the following inclusion criteria: (1) aged ≥ 18 years with group 1 pulmonary hypertension based on the 2022 ESC/ERS Guidelines criteria valid at the time; (2) undergoing a diagnostic or follow-up RHC with IF, DF, and TD data available. All other forms of PH were excluded. Patients with congenital heart disease and a shunt were excluded [21]. The patients flowchart is illustrated in the Supplementary Figure S1. Demographic, clinical, biochemical, hemodynamic, radiological, and functional data were collected and anonymized before analysis.

### 2.2. Right Heart Catheterization

The hemodynamic assessment was performed using an air-filled balloon-tipped 7F Swan–Ganz TD catheter (131F7, Edwards Lifesciences, Irvine, CA, USA). All patients underwent RHC performed by a single operator (FLG), following a standardized protocol that includes a saturation run to exclude a shunt and the measurement of right atrial, right ventricle, pulmonary artery, and wedge pressures. At the same time, breath-by-breath VO_2_ averaged over 15 min was measured using a portable machine (Jaeger Oxycon Mobile PCa, VIASYS Healthcare GmbH, Leibnizstr, Germany).

The SVI by the Fick method (DF-SVI) was calculated using the following equation:

$$\frac{VO_{2}(\mathrm{mL}\cdot \min^{-1})}{\mathrm{heart}\;\mathrm{rate}\;(\cdot \min^{-1})\;\cdot \;\mathrm{arteriovenous}\;\mathrm{oxygen}\;\mathrm{content}\;\mathrm{difference}\;(\mathrm{mL}\cdot {100\mathrm{mL}}^{-1})\;\cdot \;\mathrm{body}\;\mathrm{surface}\;\mathrm{area}\;(m^{2})}$$

The VO_2_ for the IF method (IF-SVI) was estimated using the Dehmer et al. equation, shown to be the most accurate [9].

The thermodilution SVI (TD-SVI) was obtained using a standardized technique [6]. The mean values of at least 3 measurements in sinus rhythm and 5 in atrial fibrillation with <10% variability were used.

### 2.3. Cardiac Magnetic Resonance Imaging

A subset of patients had a CMR performed within 24 h of the RHC. All CMRs were reviewed by a single radiologist (DG). The exams were performed using a 1.5 T Aera scanner (Siemens Healthcare, Erlangen, Germany), using a 32-channel phased array surface coil. Short-axis and transaxial cine images were acquired with a breath-held balanced steady-state free precession technique to derive contiguous parallel short-axis slices of both ventricles from the base to the apex. RV and LV volumes were determined by drawing the endocardial and epicardial contours manually, at end diastole and end systole, using commercially available software (Circle CVI42, version 5.12.1, Calgary, AB, Canada). For the purpose of analysis, papillary muscles were included as part of the ventricular cavity volumes. Ventricular volumes were indexed for Body Surface Area (BSA). Stroke volume was measured from the left ventricular volumes, already proven to be the most accurate compared to DF [12].

### 2.4. Echocardiography

All echocardiographic measurements were made according to the guidelines, using M-mode, 2D, pulsed-wave, continuous-wave, and color-Doppler imaging, and were performed using dedicated views [22]. Tricuspid regurgitation severity was assessed at the time of diagnosis and graded using qualitative Doppler and semiquantitative parameters (color flow jet area, intensity and shape of continuous wave Doppler, and vena contracta in the apical four-chamber view) by a single experienced echocardiographer operator during post-processing analysis and classified on an ordinal scale as absent, mild, moderate, or severe.

### 2.5. Statistics

Normality was assessed through the Kolmogorov–Smirnov test. Quantitative data are described with means and standard deviations (SDs) or medians and interquartile ranges (IQR) according to their distribution, and qualitative data with absolute frequencies and percentages. Missing data were not imputed.

Bland–Altman analysis was used to compare the degree of agreement between IF, TD, and the DF method for SVI measurement. Bias was defined as the mean value of the differences between IF, TD, and the DF method. The limits of agreement were defined as the bias ± 2 standard deviations (SDs) reported as mL/m^2^. The percentage error was calculated as $\frac{2\cdot \mathrm{SD}}{\mathrm{mean}\;\mathrm{SVI}}$.

For each SVI measure, the values obtained through the different methods were also compared by regression analysis. The correlation coefficients between different methods were determined by Spearman’s rank correlation analysis. The Wilcoxon signed-rank test was used for direct comparison between each method and DF. Intraclass correlation coefficient (ICC) estimates for inter- and intra-observer variability in SV assessment were calculated. Twenty percent of patients were randomly selected for a repeat measurement by a second investigator to determine interobserver variability; intra-observer variability was assessed with one investigator performing re-measures after a 1-month interval, as previously reported [23].

The sample size was calculated using an estimated effect size of 0.31 based on previous literature comparing DF and IF measures. We estimated a required sample size of 100 subjects, with a desired statistical power of 85% and an α error of 0.05.

*p* values < 0.05 were considered to reflect statistical significance. Based on previous literature, a median percentage error < ±20% compared to the gold standard method was considered an acceptable criterion for interchangeability [7]. Statistical tests were performed using the Statistical Package for Social Sciences (version 28.0; SPSS, Chicago, IL, USA).

## 3. Results

### 3.1. Clinical Characteristics of the Study Population

A total of 103 patients were included, with a mean (SD) age of 56 (16) years, of whom 58 (56%) were women. The majority were diagnosed with idiopathic (56%) or connective tissue disease-associated PAH (31%). The mean pulmonary artery pressure (mPAP) was 40 (14) mmHg, with a mean pulmonary artery wedge pressure of 9 (5) mmHg. Twenty (19%) patients had severe TR. Approximately three-quarters of patients had a CMR performed within 24 h of the RHC. Demographic, echocardiographic, imaging, and hemodynamic data are shown in Table 1.

### 3.2. Thermodilution Shows a Better Agreement with the Gold Standard Direct Fick than Indirect Fick

The median (IQR) IF-SVI, TD-SVI, and DF-SVI were 25.4 (19.4–32.5) mL/m^2^, 31.1 (26.7–37.7) mL/m^2^, and 31.3 (25.6–40.3) mL/m^2^, respectively.

**Figure 1:**
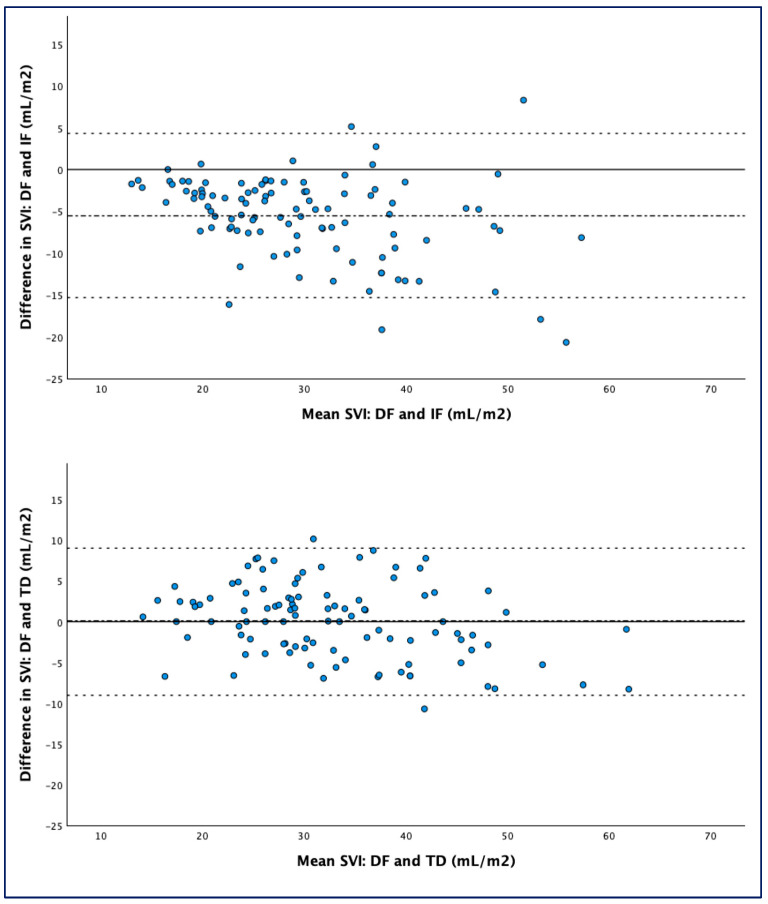
Bland–Altman plots. (**Upper panel**) shows degree of agreement between direct and indirect Fick. (**Lower panel**) shows degree of agreement between direct Fick and thermodilution. Solid line: y = 0. Dash-dotted line: mean difference (bias). Dashed lines: mean ± 2 SD (upper and lower limits of agreement). SVI indicates stroke volume index; DF, direct Fick; IF, indirect Fick; TD, thermodilution.

Differences between DF-SVI and IF-SVI and between DF-SVI and TD-SVI are shown in the Bland–Altman plots in Figure 1. Median values (IQR), mean difference (bias), limits of agreement, and percentage error according to the three different methods are reported in Table 2.

**Figure 2:**
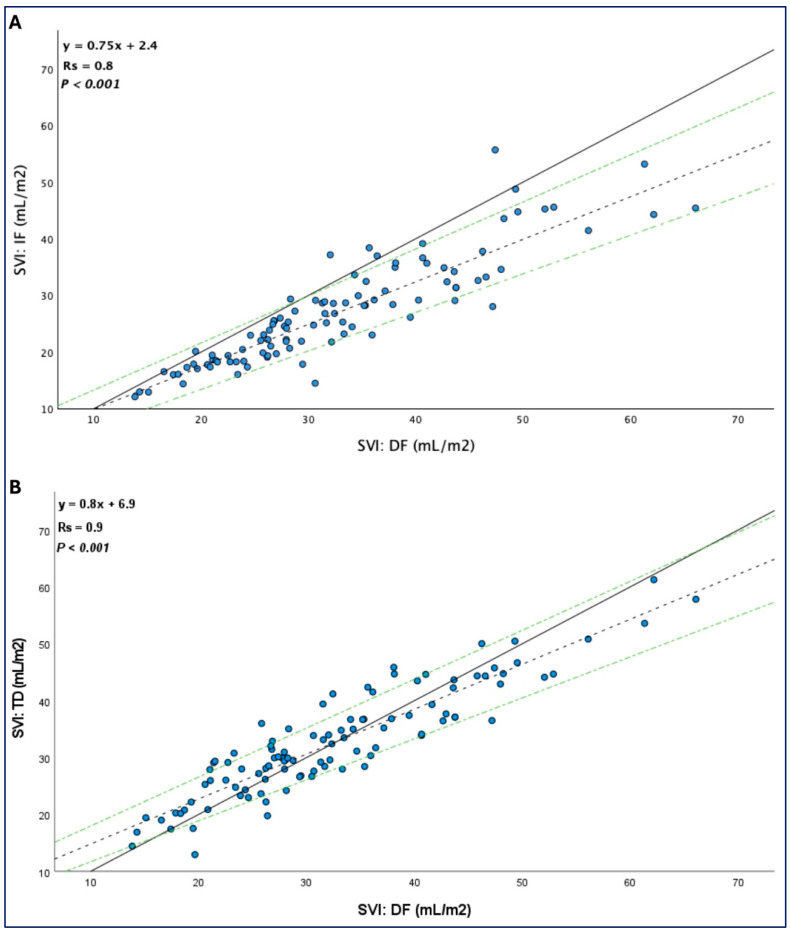
Linear regression analysis of the correlation between (**A**). Direct Fick and indirect Fick; (**B**). Direct Fick and thermodilution. The solid line indicates the line of identity (y = x); the dashed lines indicate the regression equation; green dash-dotted lines indicate a 95% confidence interval. SVI indicates stroke volume index; DF, direct Fick; IF, indirect Fick; TD, thermodilution; Rs, R square.

The mean difference, limits of agreement, percentage error, and typical error for DF-SVI vs. IF-SVI and DF-SVI vs. TD-SVI according to the degree of TR (severe vs. non-severe TR) are presented in Table 2. The overall percentage error between direct Fick and thermodilution was 10 (5–16)%, with a typical error of 3.3 mL/m^2^. No consistent directionality of error was observed between the two methods. TD-SVI showed a stronger correlation with DF-SVI than indirect Fick; the latter systematically underestimated the SVI, as shown in Figure 2.

A median percentage error of ≥20% was observed in 37 (36%) cases with IF-SVI and 17 (17%) with TD-SVI, compared to DF-SVI.

### 3.3. Low Cardiac Index and Severe Tricuspid Regurgitation Are Associated with a Greater Bias Between Thermodilution and Direct Fick

Among age, mean pulmonary artery pressure, pulmonary vascular resistance, cardiac index, and TR severity, the biggest disagreement between thermodilution and direct Fick was observed in patients with low cardiac index (*p* < 0.001), as shown in Table 2 and the Supplementary Figures S2 and S3. In patients with a CI < 2.2 L/min/m^2^ (*n* = 36), the median (IQR) SVI-DF was 22.9 (19.4–27) mL/m^2,^ and the median (IQR) SVI-TD was 26.9 mL/m^2^ (*p* = 0.007), with a median percentage error between DF and TD of 14 (6–23)% in patients with CI < 2.2 L/min/m^2^ versus 9 (5–15)% in patients with CI ≥ 2.2 L/min/m^2^.

The degree of TR severity was not linked to a greater bias by regression analysis (*p* = 0.48). However, a greater disagreement between DF and TD was observed by Bland–Altman subgroup analysis in the 20 (19%) patients with severe versus mild to moderate TR (bias 0.84 vs. −0.07, respectively), as shown in Table 2 and Supplementary Figures S4 and S5.

### 3.4. Clinical Implications of the Bias Between Measures

Risk category distribution according to DF-SVI, IF-SVI, and TD-SVI is shown in Supplementary Figure S6, based on the 2022 ESC/ERS guidelines. Using the DF-SVI, twenty-nine (28), 23 (22%), and 51 (50%) patients were in low, intermediate, and high-risk groups, respectively. Using IF-SVI, twelve (12%), 18 (18%), and 73 (70%) patients were in the low, intermediate, and high-risk groups, respectively. Using TD-SVI, twenty-five (24%), 27 (26%), and 51 (50%) were in the low, intermediate, and high-risk groups, respectively. Concordance between DF-SVI and IF-SVI risk group categories was observed in 66 (64%) patients, and between DF-SVI and TD-SVI in 79 (77%) patients.

Among the 51 patients defined as high risk by SVI-DF, 6 were reclassified as intermediate risk by thermodilution. Among the 29 patients low-risk by DF-SVI, 8 were reclassified as intermediate by TD-SVI. The difference in the proportion of low-risk (*p* = 0.388) and high-risk (*p* = 1) patients by the two methods was not statistically significant (McNemar’s test).

### 3.5. Cardiac Magnetic Resonance-Derived SVI

Seventy-seven (75%) patients underwent a cardiac magnetic resonance (CMR) within 24 h of the right heart catheterization. The median (IQR) CMR-SVI was 34 (28.5–42.8) mL/m^2^. There was an excellent inter- and intra-observer agreement for the CMR-SVI. The inter-observer ICC was 0.97 (0.90–0.99), and the intra-observer ICC was 0.97 (0.89–0.99).

**Figure 3:**
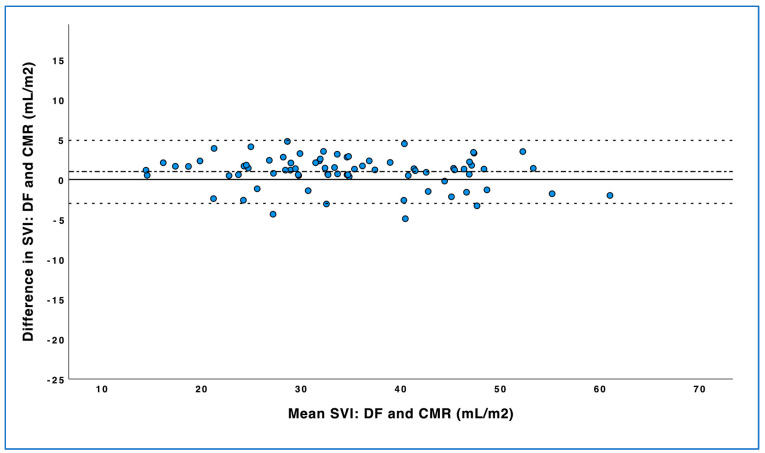
Bland–Altman plot showing the agreement between direct Fick (DF) and cardiac magnetic resonance. Solid line: y = 0. Dash-dotted line: mean difference (bias). Dashed lines: mean ± 2 SD (upper and lower limits of agreement). SVI indicates stroke volume index; DF, direct Fick; CMR, cardiac magnetic resonance.

The mean difference (bias), limits of agreement, and percentage error between DF-SVI and CMR-SVI are illustrated in Table 3. Differences between DF-SVI and CMR-SVI are shown in the Bland–Altman plots in Figure 3. Mean bias between CMR-SVI and DF-SVI was 0.99 mL/m^2^, with a median (IQR) percent error of 5 (3–9)% overall.

**Figure 4:**
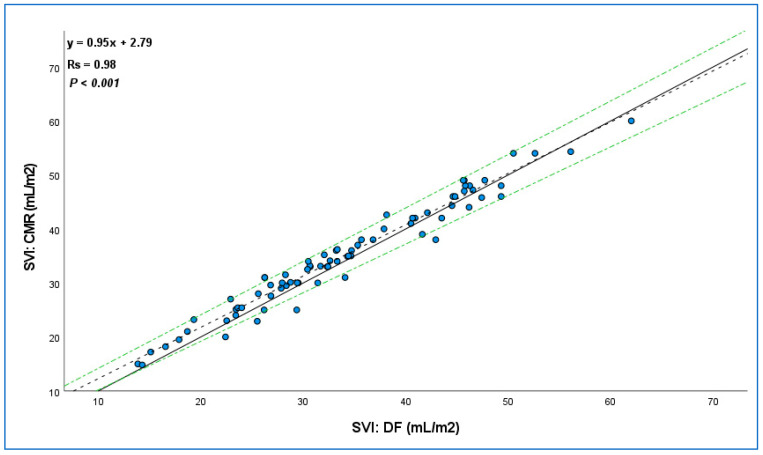
Linear regression analysis of the correlation between direct Fick and cardiac magnetic resonance. The solid line indicates the line of identity (y = x); the dashed lines indicate the regression equation; green dash-dotted lines indicate a 95% confidence interval. SVI indicates stroke volume index; DF, direct Fick; CMR, cardiac magnetic resonance.

A difference of ≥20% compared to DF-SVI was observed in only 1 patient. As shown in Figure 4, CMR-SVI showed a very strong correlation with the gold standard method (Rs 0.98). Patients with severe TR did not display a significantly greater bias than those with mild/moderate TR, as shown in Table 3 and Supplementary Figures S7 and S8, with comparable percentage errors of 5 (3–10)% and 5 (3–8)%, respectively.

## 4. Discussion

To the best of our knowledge, this is the first study aimed at assessing the agreement of alternative invasive and non-invasive methods to the gold standard direct Fick in the evaluation of stroke volume index in a well-characterized cohort of PAH patients.

The main findings can be summarized as follows: (1) The indirect Fick method is neither accurate nor precise in the estimation of SVI. (2) Thermodilution is overall an accurate and precise alternative method, with less than one-fifth showing a percentage error >20% compared to direct Fick. (3) Thermodilution is less accurate and precise in patients with severe tricuspid regurgitation and low cardiac index (i.e., CI < 2.2 L/min/m^2^). (4) CMR-derived SVI is accurate and precise, especially in patients with severe TR.

Among the four hemodynamic variables currently suggested for the risk stratification of patients with pulmonary arterial hypertension, cardiac index and stroke volume index are derived compared to the direct measurement of right atrial pressure and mixed venous oxygen saturation. Given their prognostic implications, an accurate measure of both CI and SVI is very important.

Direct Fick is the gold standard, but the VO_2_ measurement in the lab is time-consuming and not widely available. This is the reason why the indirect method, with an estimated VO_2_, is still often used. Thermodilution is an alternative method that proved to be very reliable [8,24], but data in patients with PAH, especially if associated with severe TR, are conflicting [3,4,5], and there are no comparative data regarding the SVI assessment.

Khirfan et al. recently showed that in a group of 75 PAH patients, both thermodilution and indirect Fick (only using the Dehmer equation) were accurate but not precise in the determination of CI [9].

In various clinical settings, including pulmonary hypertension, indirect Fick has proven to be less reliable than thermodilution in assessing cardiac output [14,17,18,19,20]. We confirmed this in our population, where approximately a third of cases had a percentage error >20% compared to DF, even adopting the most accurate method (the Dehmer equation) to estimate the VO_2_. 

Narang et al. reported a significant disagreement between TD and DF in the CO assessment in a large but highly heterogeneous group of patients. More than half had reduced left ventricular ejection fraction, and the median pulmonary artery wedge pressure was 18 mmHg, reflecting a significantly different population than ours focused on PAH patients. Moreover, the echocardiographic data, and especially TR assessment, was not available, and the RHC was performed over 15 years by different operators, whereas all our procedures were performed by a single operator with maximum accuracy in measurements based on current recommendations [1,5,6].

Early studies comparing TD to DF suggested the risk of CO underestimation by TD in patients with severe TR [10,25], not always confirmed in other studies [8,9,26], probably due to highly heterogeneous populations or the method of analysis used. For example, in the study of Khirfan et al., the severity of tricuspid regurgitation was not associated with an absolute difference between TD and DF in the CI estimate, but their observation was supported only by regression analysis and not a Bland–Altman subgroup analysis [9], a more accurate method to understand if the TR could have had a significant impact on the measures’ accuracy. In the study by Hoeper et al. [8], the Bland–Altman analysis showed no significant differences between DF and TD, but the subgroup of patients with severe TR was very small (*n* = 11). In our population with a higher number of patients (*n* = 20) with severe TR, the Bland–Altman analysis showed a significant bias and high percentage error.

Another point for uncertainty is whether a low cardiac output significantly impacts the accuracy of the two methods. There are limited and mixed results in the literature [8,11,15,26]. Van Grondelle et al. [11] observed a lower agreement between TD and DF in patients with a CO < 2.5 L/min, not confirmed by Hoeper et al. and Narang et al. [8,15]. Also, our data suggest that in patients with a low cardiac index, TD compared to DF overestimates the SVI. This had been previously hypothesized to be related to heat loss in low cardiac output states, with a decrease in the thermodilution curve and falsely higher computed CO [11].

The amount of data showing cardiac-magnetic resonance as a reliable non-invasive tool for prognostic stratification, also through the estimation of the SVI, is increasing [1,13,27,28,29,30]. The stroke volume measured using left ventricular volumes or aortic flow proved to be accurate compared with direct Fick [12]. The available literature provides limited data on the accuracy of CMR compared to thermodilution in the estimation of stroke volume. Early studies demonstrated good agreement between velocity-encoded, phase-difference MRI-derived cardiac output and both the DF method and TD in a small cohort of healthy volunteers [28]. However, more recent findings in a small group of patients with pulmonary hypertension suggest otherwise, showing that stroke volume measurements derived from CMR and TD did not meet criteria for interchangeability [30].

We observed that CMR left ventricular-derived SVI values were more precise than TD compared to DF, showing a significantly lower overall median percentage error of 5 (3–9)% and narrower limits of agreement. More interestingly, the degree of TR had no significant impact on either its accuracy or precision, suggesting CMR is an accurate and precise non-invasive method also in patients with severe TR.

The main limitations of our study are its monocentric nature, limiting the external validity of the results, and the small sample size for some of the subgroup analyses (e.g., the severe TR subgroup).

In conclusion, our study confirms the low reliability of the indirect Fick in measuring the SVI in PAH patients. Thermodilution is the best alternative if the direct Fick is not feasible, but it should be used with caution in patients with severe TR or low CI. SVI assessed by cardiac magnetic resonance might represent a valid non-invasive alternative because it is not biased by the presence of severe TR. Our observations need to be confirmed by larger studies.

## Figures and Tables

**Table 1:** **Baseline cohort characteristics.** All quantitative data: mean (standard deviation), unless otherwise specified. ^#^ median (interquartile range). BMI indicates body mass index; BSA, body surface area; IPAH, idiopathic pulmonary arterial hypertension; CTD-PAH, connective tissue disease associated pulmonary arterial hypertension; HPAH, hereditary pulmonary arterial hypertension; HIV-PAH, human immunodeficiency virus infection associated pulmonary arterial hypertension; PVOD/PCH, pulmonary veno-occlusive disease/pulmonary capillary hemangiomatosis; WHO-FC, World Health Organization functional class; COPD, chronic obstructive pulmonary disease; RA, right atrium; LVEF, left ventricular ejection fraction; TAPSE, tricuspid annular plane systolic excursion; TR, tricuspid regurgitation; MR, mitral regurgitation; PR, pulmonary regurgitation; AR, aortic regurgitation; RVEF, right ventricular ejection fraction; LVSVI, left ventricular stroke volume index; BP, blood pressure; RAP, right atrial pressure; PAP, pulmonary artery pressure; PAWP, pulmonary artery wedge pressure; SvO_2_, mixed venous oxygen saturation.

	N = 103
Age, years	56 (16)
Female, *n*	58 (56%)
BMI, Kg/m^2^	27.1 (6.3)
BSA, m^2 #^	1.84 (1.69–2.03)
PAH diagnosis	
IPAH	58 (56%)
CTD-PAH	32 (31%)
HPAH	7 (7%)
HIV-PAH	5 (5%)
PVOD/PCH	1 (1%)
WHO-FC 1/2/3/4	4/32/63/4
**Comorbidities**	
Systemic arterial hypertension	39 (38%)
Ischemic heart disease	12 (12%)
Atrial fibrillation	14 (14%)
COPD	17 (17%)
Chronic kidney disease	10 (10%)
Type 2 Diabetes mellitus	19 (18%)
**Echocardiography**	
RA area indexed, cm^2^/m^2 #^	11.9 (9.5–14.8)
LVEF, %	59 (4)
TAPSE, mm	17 (5)
TR 0/1/2/3	0/47/36/20
MR 0/1/2/3	71/27/5/0
PR 0/1/2/3	77/23/3/0
AR 0/1/2/3	98/4/1/0
**Cardiac magnetic resonance imaging (*n* = 77)**	
Heart rate, beats/min	77 (14)
RVEF, %	40 (13)
LVSVI, mL/m^2 #^	34 (28.5–42.8)
**Right heart catheterization**	
Heart rate, beats/min	79 (14)
Mean systemic BP, mmHg	86 (14)
Measured VO_2_, mL/min	246 (49)
Estimated VO_2_, mL/min	205 (43)
Mean RAP, mmHg	7 (4)
Mean PAP, mmHg	40 (14)
Mean PAWP, mmHg	9 (5)
SvO_2_, %	62 (10)

**Table 2:** **Results of Bland–Altman analysis.** Bland–Altman analysis of stroke volume index measured by indirect Fick and thermodilution compared to direct Fick overall and stratified by the degree of TR and CI. IF indicates indirect Fick; TD, thermodilution; SVI, stroke volume index; TR, tricuspid regurgitation; CI, cardiac index; IQR, interquartile range.

	Median (IQR)	Bias	Limits of Agreement	Median Percentage Error (IQR)	Typical Error	*p* (vs. DF-SVI)
IF-SVI	25.4 (19.4–32.5)	−5.53	−15.3 to 4.3	15 (9–25)	3.5	<0.001
TD-SVI	31.1 (26.7–37.7)	0.09	−9.01 to 9.01	10 (5–16)	3.3	0.861
TD-SVI (Mild/moderate TR) *n* = 83	32.4 (27.9–41.2)	−0.07	−9.1 to 8.9	10 (5–16)	3.2	0.901
TD-SVI (Severe TR) *n* = 20	28 (22.4–35.5)	0.84	−8.66 to 10.1	14 (7–17)	3.4	0.573
TD-SVI (CI ≥ 2.2 L/min/m^2^) *n* = 67	36.4 (29.9–43.7)	−0.9	−10.1 to 8.3	9 (5–15)	3.3	0.107
TD-SVI (CI < 2.2 L/min/m^2^) *n* = 36	26.9 (20.4–29.5)	1.9	−5.9 to 9.7	14 (6–23)	2.8	0.007

**Table 3:** **Results of Bland–Altman analyses of CMR based measurements for stroke volume index.** Bland–Altman analysis of cardiac magnetic resonance measures of stroke volume index, overall and based on TR degree. SVI indicates stroke volume index; DF, direct Fick; CMR, cardiac magnetic resonance.

	Median (IQR)	Bias	Limits of Agreement	Median Percent Error (IQR)	Typical Error	*p* (vs. DF-SVI)
CMR-SVI	34 (28.5–42.8)	0.99	−3.09 to 5.07	5 (3–9)	1.5	<0.001
CMR-SVI (Mild/moderate TR) *n* = 62	35.1 (30–45.9)	0.92	−3.24 to 5.08	5 (3–8)	1.5	0.002
CMR-SVI (Severe TR) *n* = 15	27 (23.2–34.1)	1.13	−2.55 to 5.21	5 (3–10)	1.4	0.011

## Data Availability

The original contributions presented in this study are included in the article/supplementary material. Further inquiries can be directed to the corresponding author.

## Supplementary Materials

The following supporting information can be downloaded at: https://www.mdpi.com/article/10.3390/life15010054/s1, Supplementary Figure S1. Patients flowchart; Supplementary Figure S2. Bland-Altman plots showing degree of agreement between direct Fick and thermodilution: patients stratified according to CI; Supplementary Figure S3. Linear regression analysis of the correlation between direct Fick and thermodilution for SVI: patients stratified according to CI; Supplementary Figure S4. Bland-Altman plots showing degree of agreement between direct Fick and thermodilution: patients stratified according to the degree of tricuspid regurgitation; Supplementary Figure S5. Linear regression analysis of the correlation between direct Fick and thermodilution for SVI: patients stratified according to the degree of tricuspid regurgitation; Supplementary Figure S6. Sankey diagrams showing change in risk status according to DF-SVI, TD-SVI and IF-SVI; Supplementary Figure S7. Bland-Altman plots showing degree of agreement between direct Fick and cardiac magnetic resonance: patients stratified according to the degree of tricuspid regurgitation; Supplementary Figure S8. Linear regression analysis of the correlation between direct Fick and cardiac magnetic resonance for SVI: patients stratified according to the degree of tricuspid regurgitation.

## References

[1] Humbert M., Kovacs G., Hoeper M.M., Badagliacca R., Berger R.M.F., Brida M., Carlsen J., Coats A.J.S., Escribano-Subias P., Ferrari P. (2023). 2022 ESC/ERS Guidelines for the diagnosis and treatment of pulmonary hypertension. Eur. Respir. J..

[2] Weatherald J., Boucly A., Chemla D., Savale L., Peng M., Jevnikar M., Jaïs X., Taniguchi Y., O’connell C., Parent F. (2018). Prognostic Value of Follow-Up Hemodynamic Variables After Initial Management in Pulmonary Arterial Hypertension. Circulation.

[3] Boucly A., Beurnier A., Turquier S., Jevnikar M., de Groote P., Chaouat A., Cheron C., Jaïs X., Picard F., Prévot G. (2024). Risk stratification refinements with inclusion of haemodynamic variables at follow-up in patients with pulmonary arterial hypertension. Eur. Respir. J..

[4] Dardi F., Boucly A., Benza R., Frantz R., Mercurio V., Olschewski H., Rådegran G., Rubin L.J., Hoeper M.M. (2024). Risk stratification and treatment goals in pulmonary arterial hypertension. Eur. Respir. J..

[5] D’alto M., Dimopoulos K., Coghlan J.G., Kovacs G., Rosenkranz S., Naeije R. (2018). Right Heart Catheterization for the Diagnosis of Pulmonary Hypertension: Controversies and Practical Issues. Heart Fail. Clin..

[6] Rosenkranz S., Preston I.R. (2015). Right heart catheterisation: Best practice and pitfalls in pulmonary hypertension. Eur. Respir. Rev..

[7] Genecand L., Adler D., Beghetti M., Lador F. (2021). Cardiac Output Determination in Precapillary Pulmonary Hypertension: A Systematic Review. Respiration.

[8] Hoeper M.M., Maier R., Tongers J., Niedermeyer J., Hohlfeld J.M., Hamm M., Fabel H. (1999). Determination of Cardiac Output by the Fick Method, Thermodilution, and Acetylene Rebreathing in Pulmonary Hypertension. Am. J. Respir. Crit. Care Med..

[9] Khirfan G., Ahmed M.K., Almaaitah S., Almoushref A., Agmy G.M., Dweik R.A., Tonelli A.R. (2019). Comparison of Different Methods to Estimate Cardiac Index in Pulmonary Arterial Hypertension. Circulation.

[10] Cigarroa R.G., Lange R.A., Williams R.H., Bedotto J.B., Hillis L.D. (1989). Underestimation of cardiac output by thermodilution in patients with tricuspid regurgitation. Am. J. Med..

[11] van Grondelle A., Ditchey R.V., Groves B.M., Wagner W.W., Reeves J.T. (1983). Thermodilution method overestimates low cardiac output in humans. Am. J. Physiol. Circ. Physiol..

[12] Mauritz G.-J., Marcus J.T., Boonstra A., Postmus P.E., Westerhof N., Vonk-Noordegraaf A. (2008). Non-invasive stroke volume assessment in patients with pulmonary arterial hypertension: Left-sided data mandatory. J. Cardiovasc. Magn. Reson..

[13] Alabed S., Shahin Y., Garg P., Alandejani F., Johns C.S., Lewis R.A., Condliffe R., Wild J.M., Kiely D.G., Swift A.J. (2021). Cardiac-MRI Predicts Clinical Worsening and Mortality in Pulmonary Arterial Hypertension: A Systematic Review and Meta-Analysis. JACC Cardiovasc. Imaging.

[14] Genecand L., Simian G., Desponds R., Wacker J., Ulrich S., Lechartier B., Fellrath J.-M., Sitbon O., Beghetti M., Lador F. (2023). The Influence of Methods for Cardiac Output Determination on the Diagnosis of Precapillary Pulmonary Hypertension: A Mathematical Model. J. Clin. Med..

[15] Narang N., Thibodeau J.T., Parker W.F., Grodin J.L., Garg S., Tedford R.J., Levine B.D., McGuire D.K., Drazner M.H. (2022). Comparison of Accuracy of Estimation of Cardiac Output by Thermodilution Versus the Fick Method Using Measured Oxygen Uptake. Am. J. Cardiol..

[16] Desole S., Obst A., Habedank D., Opitz C.F., Knaack C., Hortien F., Heine A., Stubbe B., Ewert R. (2022). Comparison between thermodilution and Fick methods for resting and exercise-induced cardiac output measurement in patients with chronic dyspnea. Pulm. Circ..

[17] Fares W.H., Blanchard S.K., Stouffer G.A., Chang P.P., Rosamond W.D., Ford H.J., Aris R.M. (2012). Thermodilution and fick cardiac outputs differ: Impact on pulmonary hypertension evaluation. Can. Respir. J..

[18] Alkhodair A., Tsang M.Y., Cairns J.A., Swiston J.R., Levy R.D., Lee L., Huckell V.F., Brunner N.W. (2018). Comparison of thermodilution and indirect Fick cardiac outputs in pulmonary hypertension. Int. J. Cardiol..

[19] Opotowsky A.R., Hess E., Maron B.A., Brittain E.L., Barón A.E., Maddox T.M., Alshawabkeh L.I., Wertheim B.M., Xu M., Assad T.R. (2017). Thermodilution vs Estimated Fick Cardiac Output Measurement in Clinical Practice: An Analysis of Mortality From the Veterans Affairs Clinical Assessment, Reporting, and Tracking (VA CART) Program and Vanderbilt University. JAMA Cardiol..

[20] Volodarsky I., Kerzhner K., Haberman D., Cuciuc V., Poles L., Blatt A., Kirzhner E., George J., Gandelman G. (2023). Comparison between Cardiac Output and Pulmonary Vascular Pressure Measured by Indirect Fick and Thermodilution Methods. J. Pers. Med..

[21] Nishikawa T., Dohi S. (1993). Errors in the measurement of cardiac output by thermodilution. Can. J. Anaesth..

[22] Augustine D.X., Coates-Bradshaw L.D., Willis J., Harkness A., Ring L., Grapsa J., Coghlan G., Kaye N., Oxborough D., Robinson S. (2018). Echocardiographic assessment of pulmonary hypertension: A guideline protocol from the British Society of Echocardiography. Echo Res. Pract..

[23] Leong K., Howard L., Giudice F.L., Davies R., Haji G., Gibbs S., Gopalan D. (2023). Utility of cardiac magnetic resonance feature tracking strain assessment in chronic thromboembolic pulmonary hypertension for prediction of REVEAL 2.0 high risk status. Pulm. Circ..

[24] Fegler G. (1957). The reliability of the thermodilution method for determination of the cardiac output and the blood flow in central veins. Q. J. Exp. Physiol. Cogn. Med. Sci..

[25] Konishi T., Nakamura Y., Morii I., Himura Y., Kumada T., Kawai C. (1992). Comparison of thermodilution and fick methods for measurement of cardiac output in tricuspid regurgitation. Am. J. Cardiol..

[26] Gonzalez J., Delafosse C., Fartoukh M., Capderou A., Straus C., Zelter M., Derenne J.-P., Similowski T. (2003). Comparison of bedside measurement of cardiac output with the thermodilution method and the Fick method in mechanically ventilated patients. Crit. Care.

[27] McLure L.E.R., Peacock A.J. (2009). Cardiac magnetic resonance imaging for the assessment of the heart and pulmonary circulation in pulmonary hypertension. Eur. Respir. J..

[28] Hundley W.G., Li H.F., Hillis L.D., Meshack B.M., Lange R.A., Willard J.E., Landau C., Peshock R.M. (1995). Quantitation of cardiac output with velocity-encoded, phase-difference magnetic resonance imaging. Am. J. Cardiol..

[29] Po J.R., Tong M., Meeran T., Potluri A., Raina A., Doyle M., Biederman R. (2020). Quantification of Cardiac Output with Phase Contrast Magnetic Resonance Imaging in Patients with Pulmonary Hypertension. J. Clin. Imaging Sci..

[30] Crowe L.A., Genecand L., Hachulla A.-L., Noble S., Beghetti M., Vallée J.-P., Lador F. (2022). Non-Invasive Cardiac Output Determination Using Magnetic Resonance Imaging and Thermodilution in Pulmonary Hypertension. J. Clin. Med..

