# Supplementary material for: How to Reliably Measure Stroke Volume Index in Pulmonary Arterial Hypertension: A Comparison of Thermodilution, Direct and Indirect Fick, and Cardiac MRI
Source: Life (Basel). 2025 Jan 3;15(1):54. doi: 10.3390/life15010054 (PMC11766935; doi:10.3390/life15010054)

SUPPLEMENTARY MATERIAL

Supplementary Figure S1. Patients flowchart

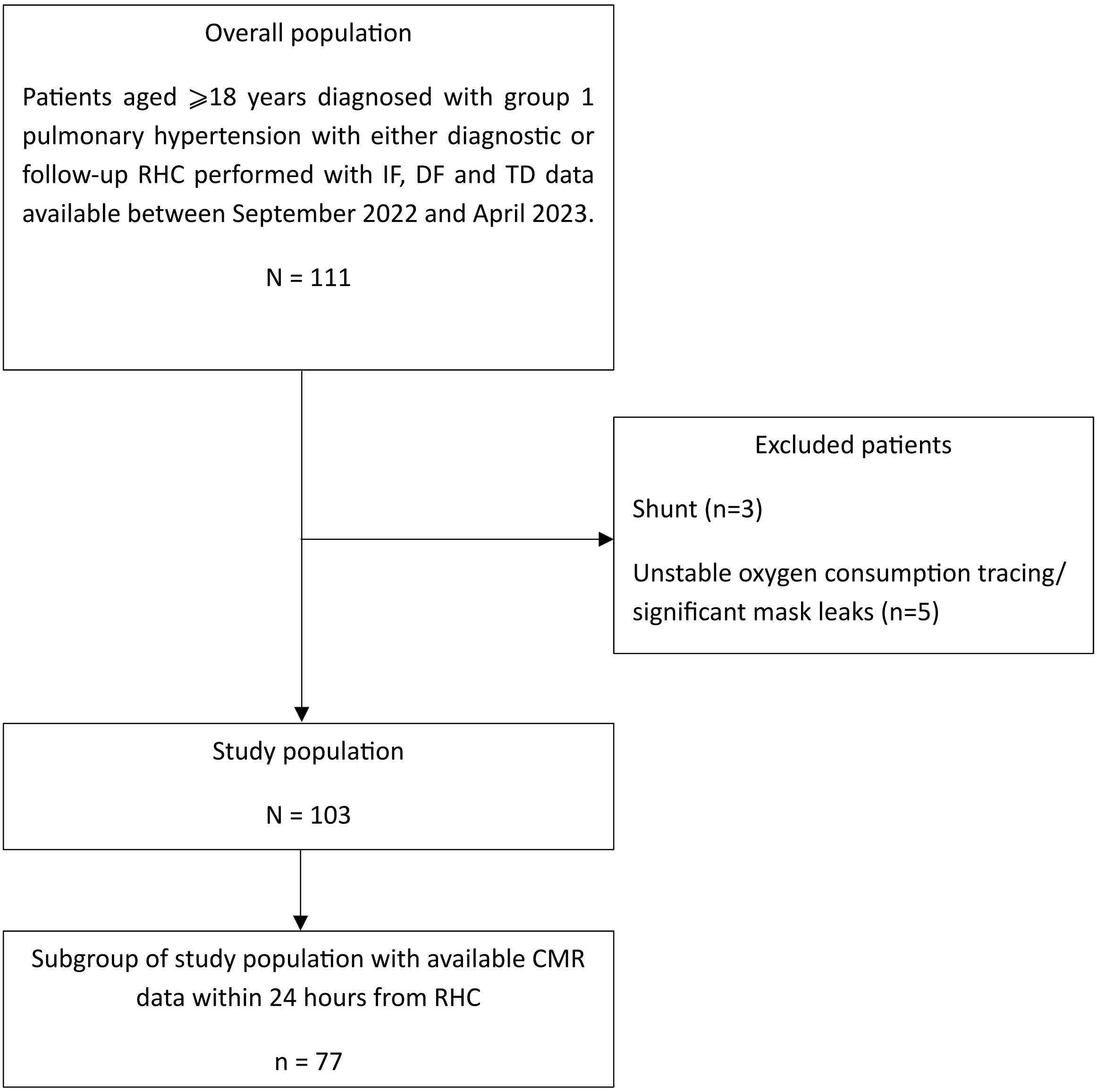

Supplementary Figure S2. Bland-Altman plots showing degree of agreement between direct Fick and thermodilution: patients stratified according to CI.

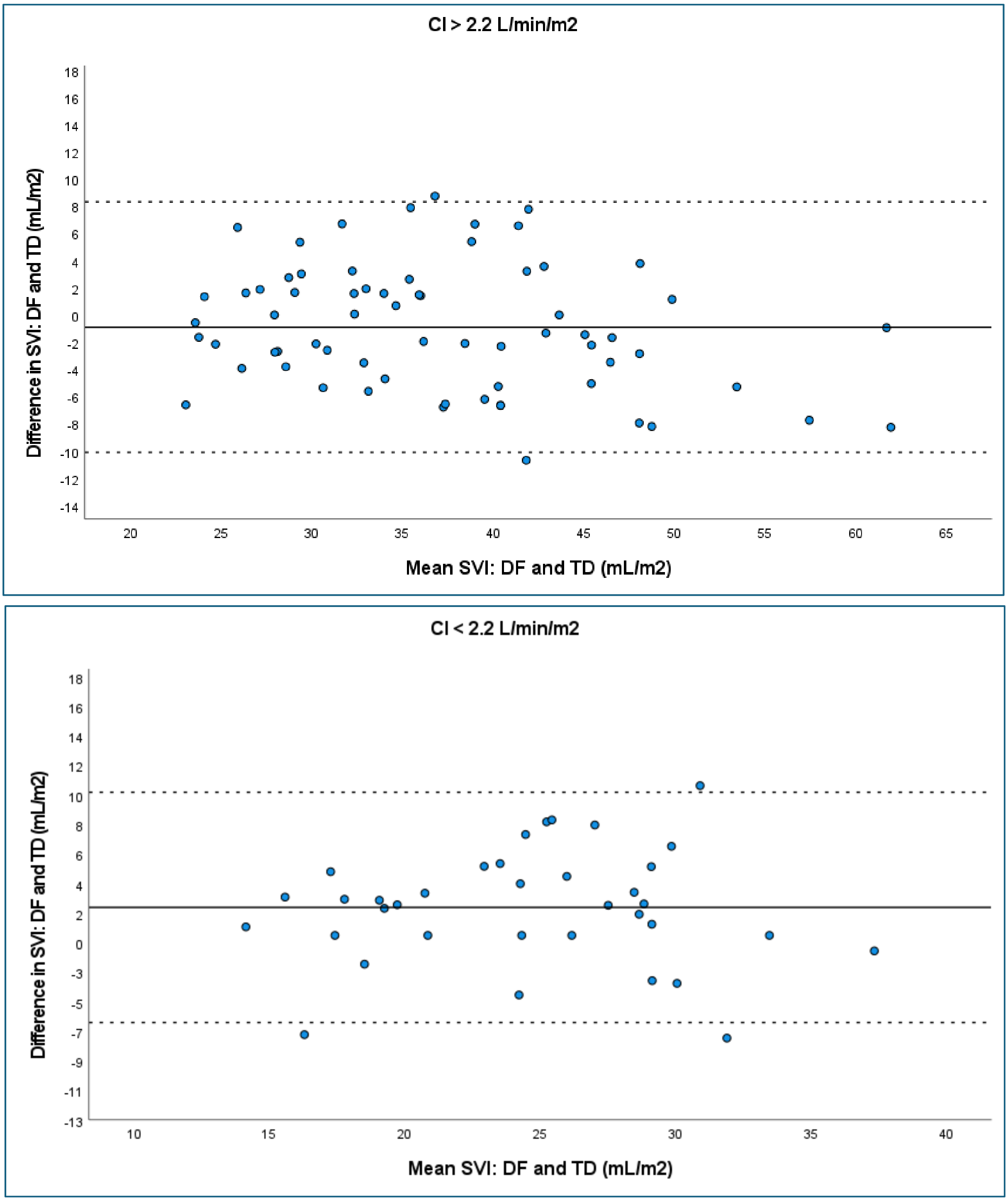

Supplementary Figure S3. Linear regression analysis of the correlation between direct Fick and thermodilution for SVI: patients stratified according to CI.

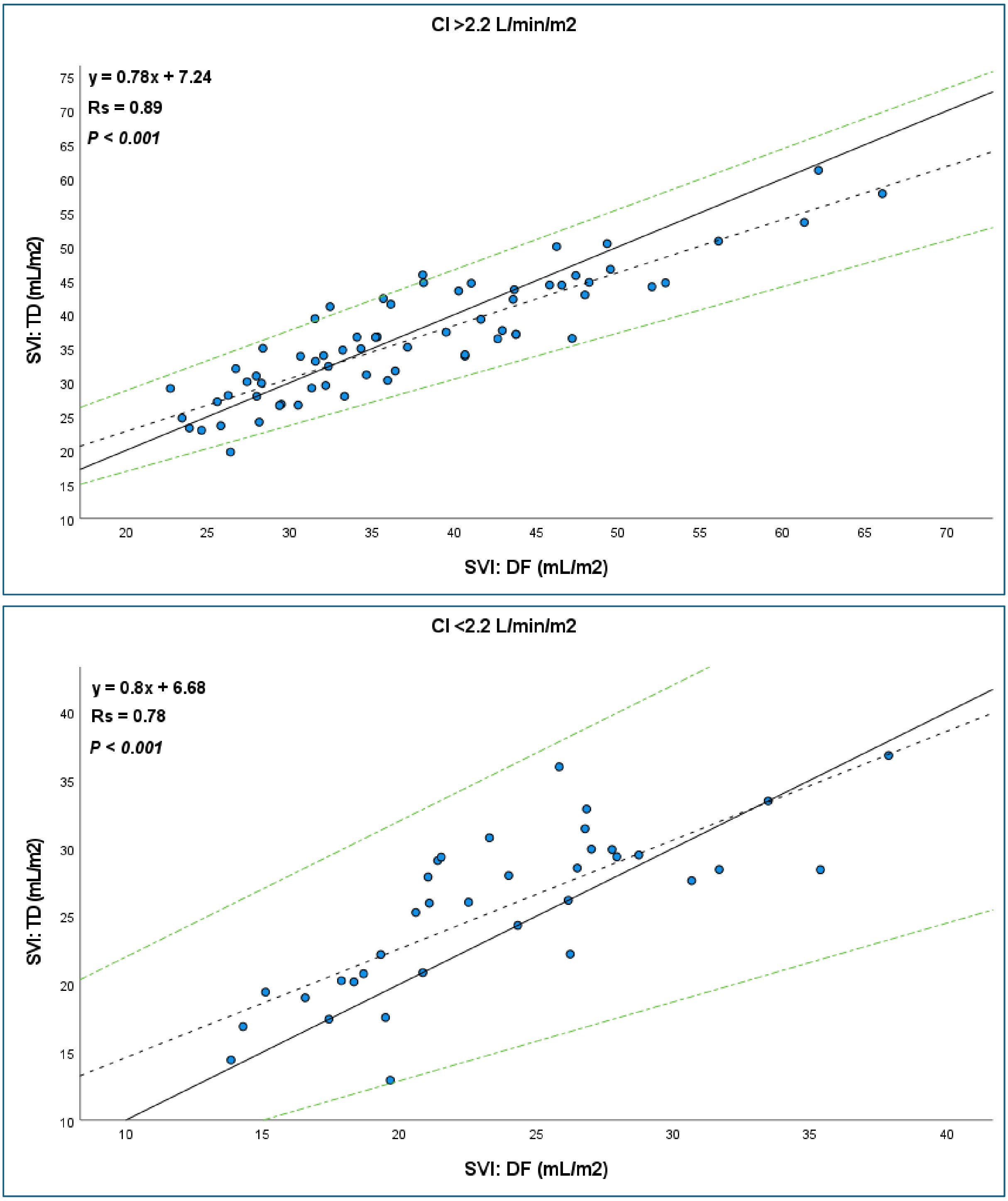

solid line: line of identity (y=x). Dashed line: regression equation. Dash-dotted green lines: 95% CI

Supplementary Figure S4. Bland-Altman plots showing degree of agreement between direct Fick and thermodilution: patients stratified according to the degree of tricuspid regurgitation.

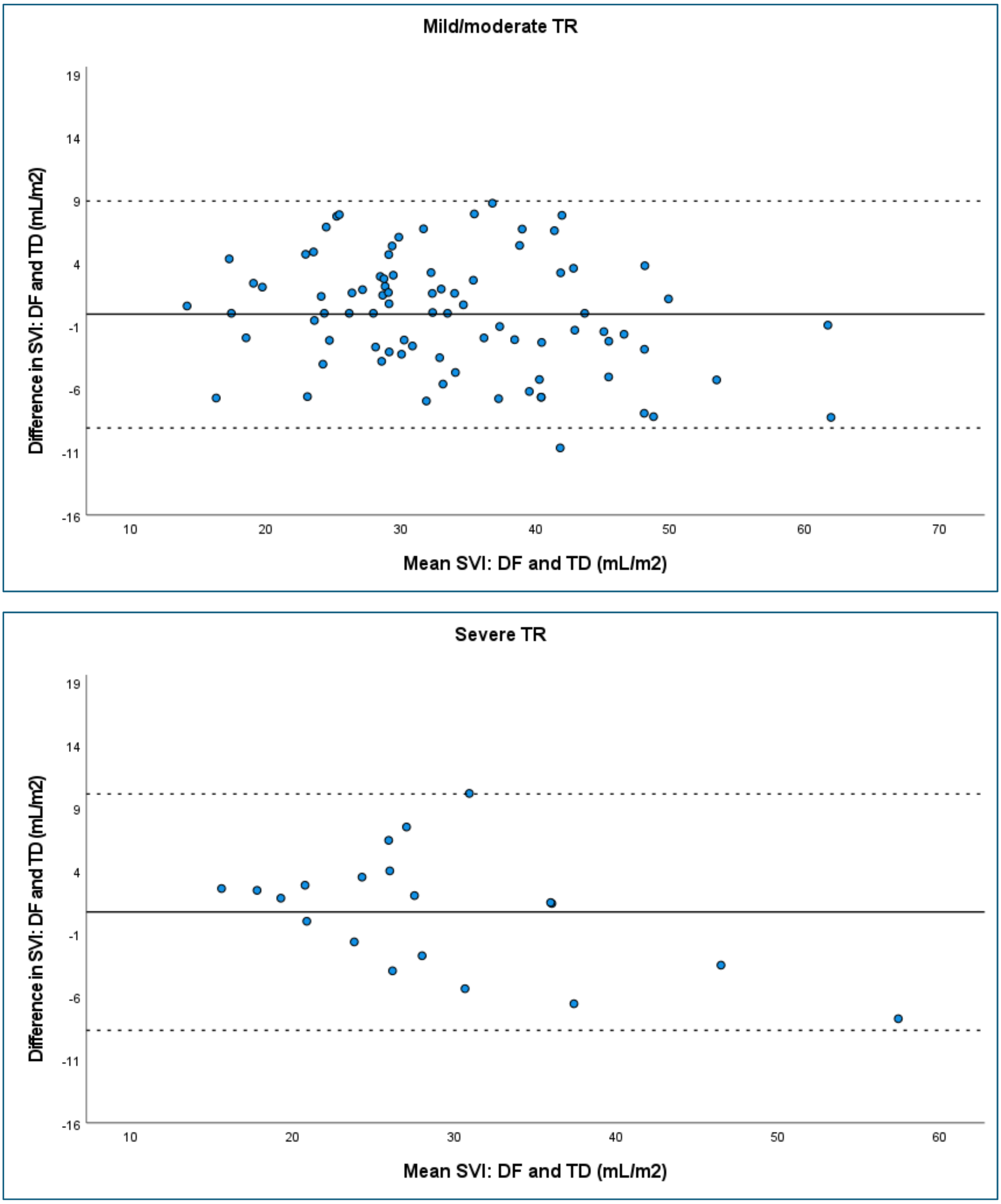

Supplementary Figure S5. Linear regression analysis of the correlation between direct Fick and thermodilution for SVI: patients stratified according to the degree of tricuspid regurgitation

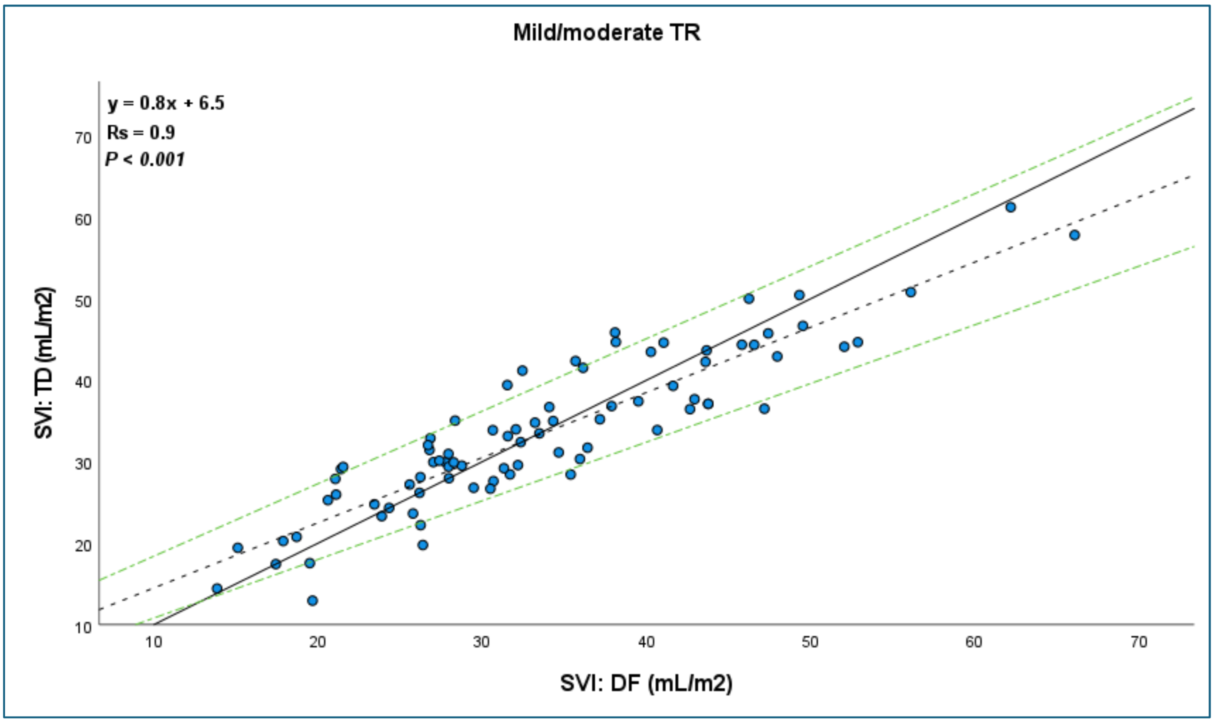

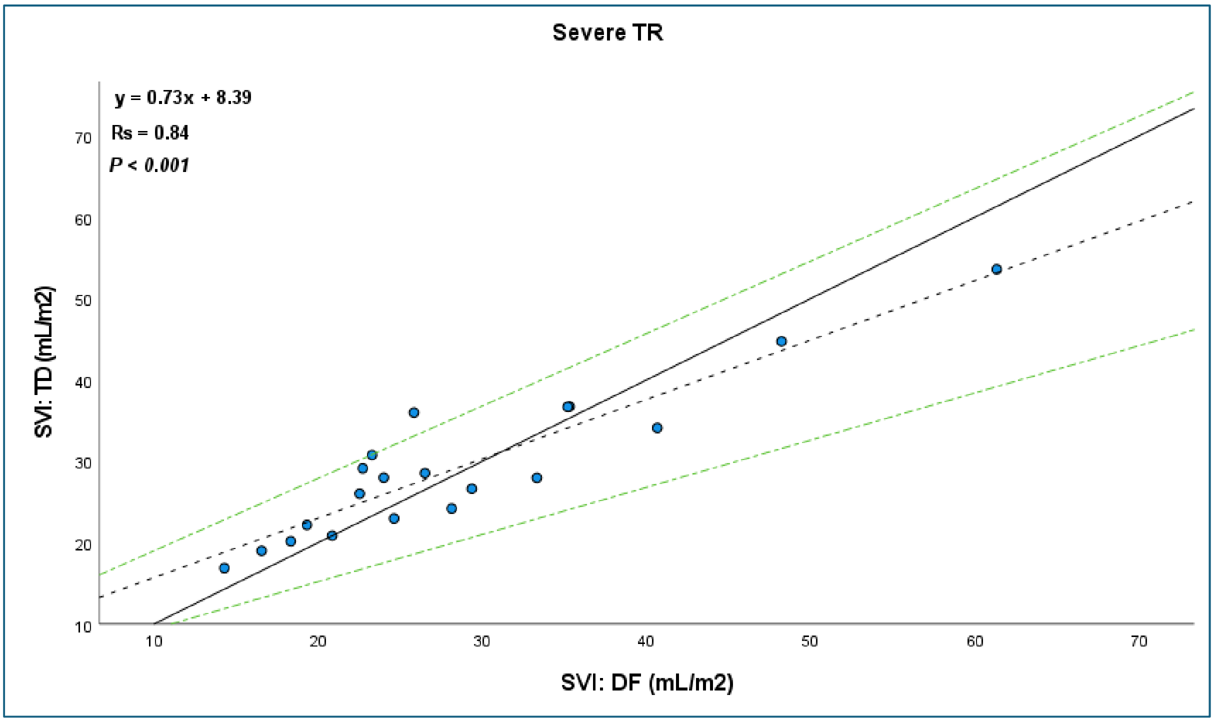

solid line: line of identity (y=x). Dashed line: regression equation. Dash-dotted green lines: 95% CI

Supplementary Figure S6. Sankey diagrams showing change in risk status according to DF-SVI, TD-SVI and IF-SVI.

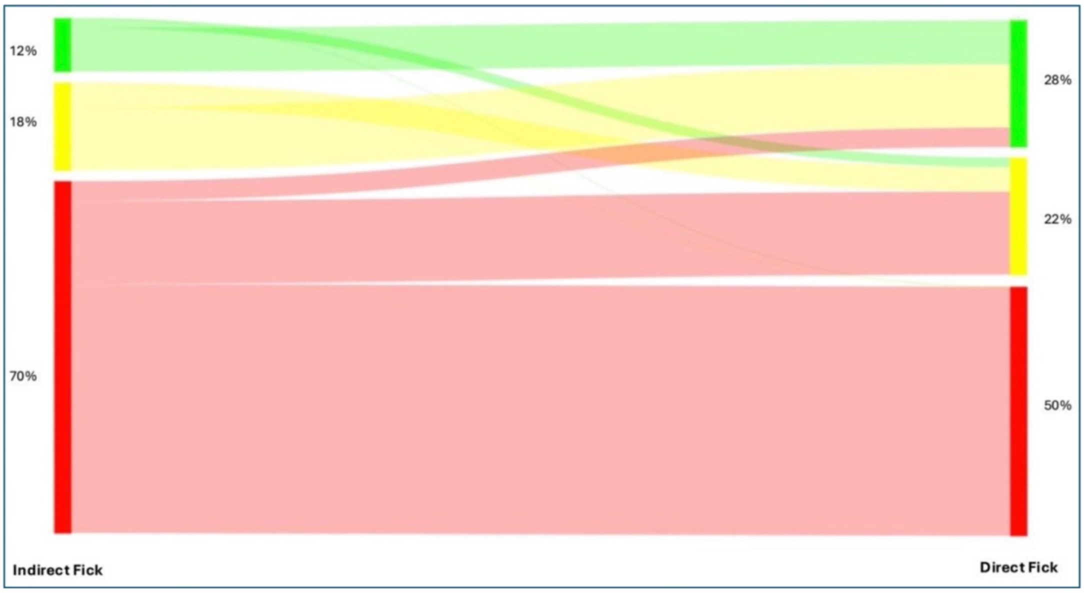

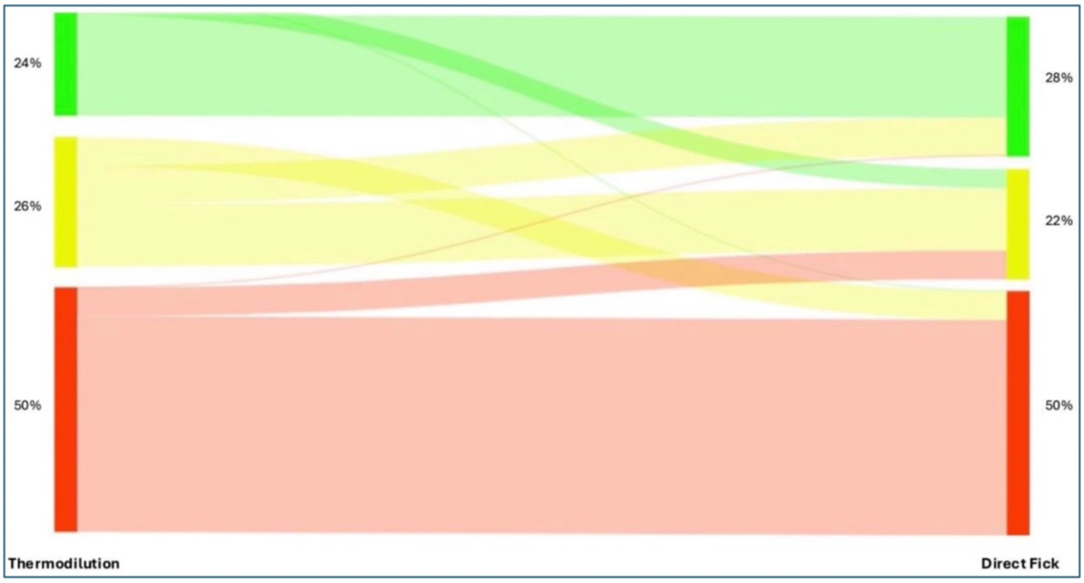

Supplementary Figure S7. Bland-Altman plots showing degree of agreement between direct Fick and cardiac magnetic resonance: patients stratified according to the degree of tricuspid regurgitation.

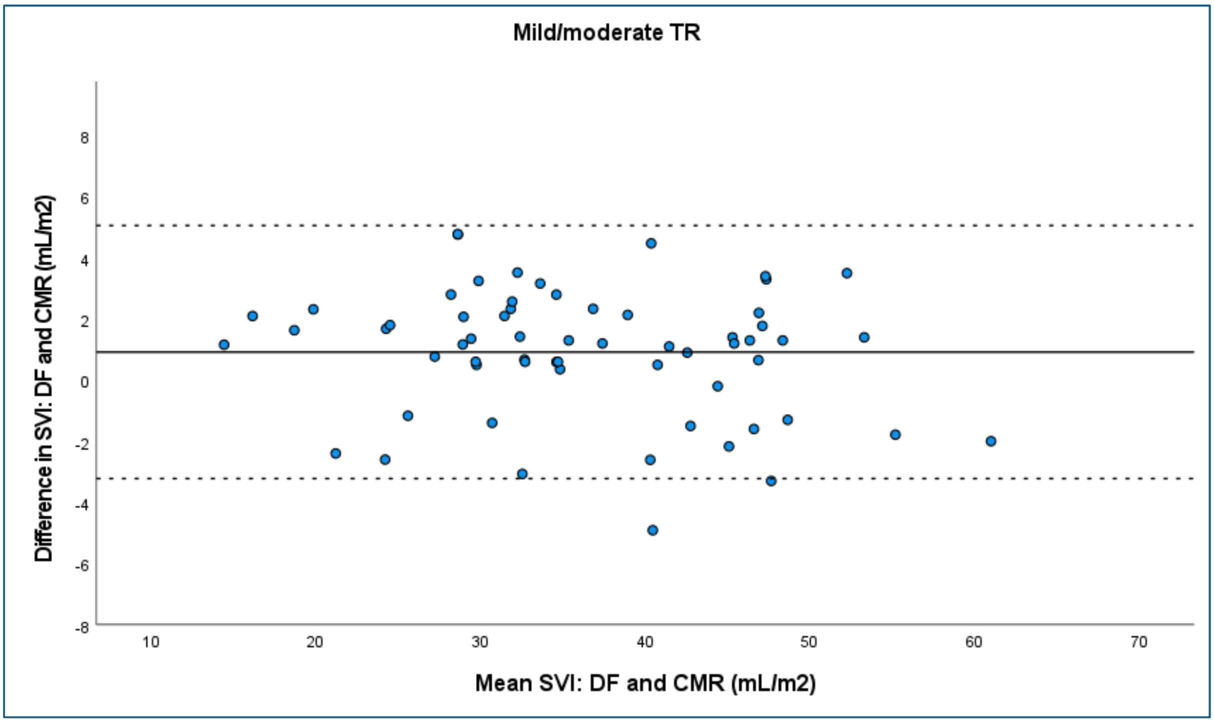

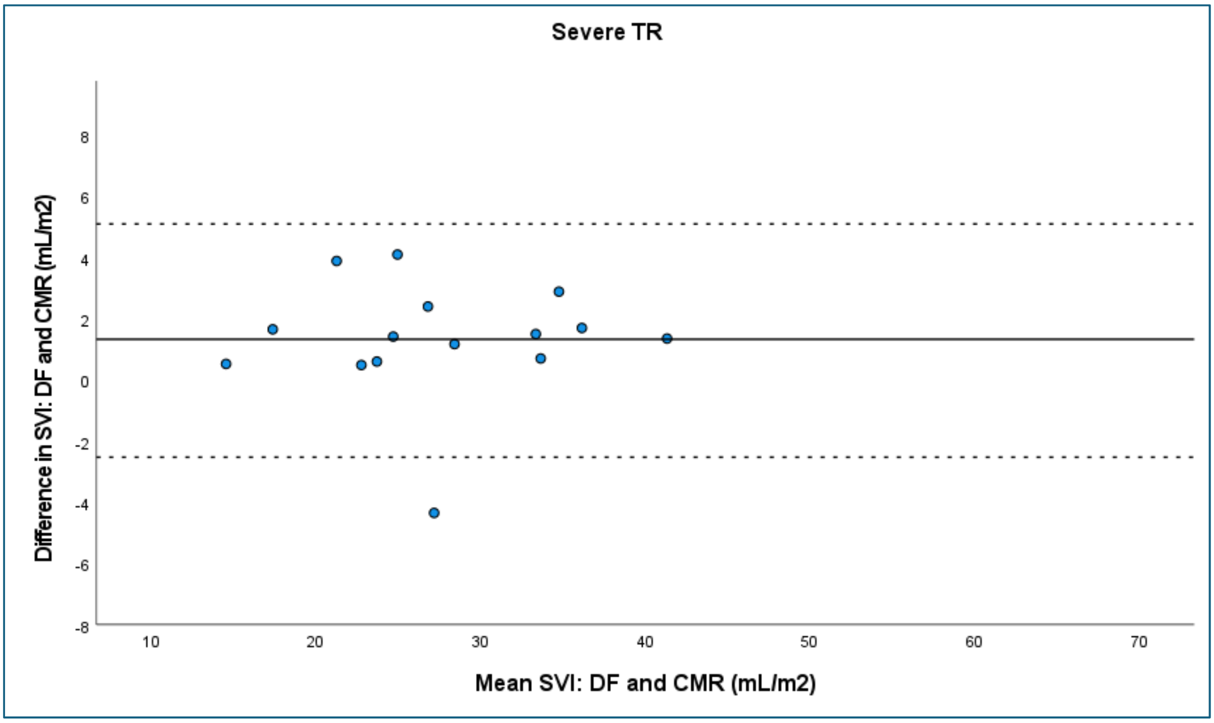

Supplementary Figure S8. Linear regression analysis of the correlation between direct Fick and cardiac magnetic resonance for SVI: patients stratified according to the degree of tricuspid regurgitation

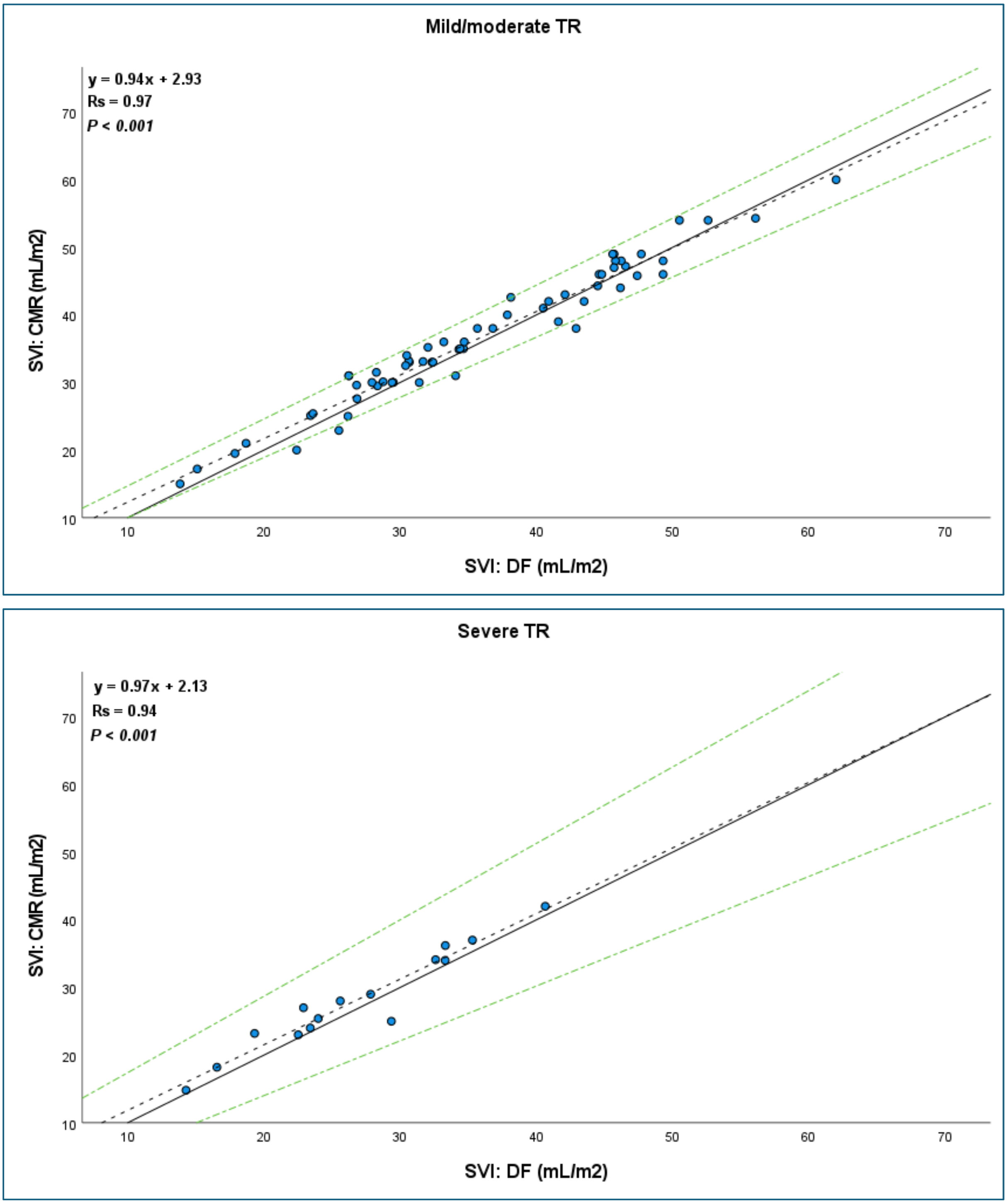

Supplement: Supplementary file 1 [file life-15-00054-s001.zip › life-3408294-supplementary.pdf]
